# What Should a Spanish Graduate in Medicine in the European Higher Education System Know About Biochemistry and Molecular Biology? A Personal Reflection

**DOI:** 10.1007/s40670-019-00721-5

**Published:** 2019-03-19

**Authors:** Pilar Morata

**Affiliations:** Area of Biochemistry and Molecular Biology, Faculty of Medicine, University of Malaga, 29071 Malaga, Spain

**Keywords:** Biochemistry and molecular biology, Degree of knowledge, Knowledge, Graduate in medicine

## Abstract

For over 40 years of working in the area of biochemistry and molecular biology, 30 of which have been spent teaching at the University of Malaga, I have been involved in the theoretical and practical teaching of the subject initially called biochemistry and now called biochemistry and molecular biology (BMB). The expansion of this scientific discipline and the renewed interest in research have led to such a large accumulation of knowledge (knowledge base) that the elaboration of a BMB program requires applying notable doses of synthesis; otherwise, it would be impossible to cover all this subject within the strict confines of the bi-semestral term. Advances in medicine and BMB are inseparable, and much of modern medicine would not be practiced as it is if it were not for our understanding of how hereditary, pathogenic, and environmental factors affect the human body at the molecular level. The importance, therefore, of teaching medical students biochemistry is evident. BMB is a subject that corresponds to the area of biomedicine and is taught during the first year of medicine. The main aim is to study the basics of chemical structures from the molecular viewpoint, with special emphasis on regulating and integrating aspects, necessary to understand such disciplines as physiology, pharmacology, or pathology. Thus, based on many years of experience teaching BMB to medical undergraduates, my aim is to define what it is that a graduate in medicine should know about the subject.

## Introduction

Biochemistry and molecular biology (BMB) really refers to the *chemistry of life*, though it overlaps and complements other disciplines, including cell biology, genetics, immunology, microbiology, pharmacology, and physiology. But precisely because of this, it runs the risk of disappearing as a separate subject due to attenuation in other disciplines. Nowadays, any research undertaken in the field of the biological or biomedical sciences includes biochemical aspects. Indeed, many fields now use the forename molecular to define the type of science involved, such as molecular genetics, molecular microbiology, molecular physiology, molecular pharmacology, and even molecular pathology. The rapid adoption of this forename by these scientific fields is actually depleting the very contents of this area of knowledge, and it is now fairly, if not, very common for these contents to be dealt with by other related areas of knowledge [1].

What really characterizes and defines BMB is that it is principally dedicated to a specific, very limited number of questions: (i) What are the three-dimensional chemical structures of biological molecules? (ii) How do these molecules interact with each other? (iii) How do cells synthesize and degrade biological molecules? (iv) How do cells conserve and use energy? (v) What are the mechanisms that organize biological molecules and coordinate their activities? (vi) How is genetic information stored, transmitted, and expressed? In addition to its obvious implications for human health, BMB reveals how the natural world functions and enables us to comprehend and appreciate the singular and mysterious condition we call *life*.

Some students consider that BMB is a fruitless exercise of memory with no transcendence for their professional future. Ironically, many students of medicine, and even practicing physicians, consider that learning biochemistry is unnecessary and that this subject is of little relevance in everyday medical tasks; accordingly, they see no use for its application in clinical practice. This utilitarian concept is, in my opinion and that of others, partly responsible for the tendency of those planning medical studies to devalue basic science and emphasize almost exclusively learning experiences related to the clinical faculty [2, 3]. However, modern biochemistry is rather more than just a catalog of molecular structures and chemical reactions. The chemistry of life has principles and concepts, eloquently described by Lehninger [4] as the “molecular logic of living organisms,” among which is man. Nevertheless, the learning and familiarization with the field of BMB requires, as Epp indicates [5], a certain degree of memorization, for example, to learn the intermediary metabolic pathways. Studying and memorizing a metabolic pathway is to acquire understanding about the strategy used by cells as a basic component of an organ (heart, lung, brain, etc.) to achieve a particular purpose. The paths must be taught with emphasis on the general principles and concepts. However, it is not necessary or desirable, or even possible, to teach all the pathways of metabolism. Teachers must choose how many pathways to teach and which ones. The decision will depend on the type of course, its level, and how much to teach. It is better to “teach fewer pathways in greater detail than to risk obscuring the forest with trees” [5].

The aim of this paper is to define how much future medical professionals should learn about BMB in general for the degree of medicine. I believe they should understand the structural elements of the various biomolecules comprising the molecular design of life, the molecular interactions and transformations produced in the different transduction pathways and storage of metabolic energy, and the regulation and integration of these pathways, as well as study the structure and metabolism of those molecules of life that transmit information.

## Biochemistry and Molecular Biology and Its Relation to Modern Medicine

The teaching and learning of BMB for undergraduates in medicine, whose levels of basic knowledge can differ greatly, have become a challenge. Students of medicine or nursing, or other health-related courses, mistakenly consider the objectives of learning biochemistry nowadays to be far removed from their future interests and needs. How to plan the classes to make them enjoyable and also how to evaluate correctly what the students learn are things all teachers have to consider, even though it is not easy. Understanding the phenomena and integrating the concepts are obstacles to be overcome and require the work and consideration of teachers and the interest and detailed study from the students.

Biochemistry is rich in facts, which need to be transmitted to the students. But more important than simply communicating the facts is that the students understand the chemical sense that governs life and how it works deficiently during pathological processes. Likewise, the subject of biochemistry imparts the basis and understanding of techniques and approaches used to solve biochemical problems. These include the action of new drugs like antidepressants, drugs used to treat diabetes, hypertension, and heart failure, and lipid-lowering drugs. BMB helps us to understand the clinical applications of recombinant proteins, viral vectors, and “omics” fields of study. It also contributes to understanding the influence of diet and lifestyle on health and how the organism ages. It describes how the cellular systems of communication are related with the response to endogenous and environmental stress. It incorporates the great progress made in recent years understanding human genetics related to emerging fields like nutrigenomics and pharmacogenomics, in the expectation that these will form the basis for the development of new personalized treatments according to each person’s individual genetic composition.

Accordingly, BMB is studied to be able to understand the relation between the molecular and structural components of nutrition (proteins, carbohydrates, and lipids; vitamins and essential minerals), metabolism (sophisticated molecular structure of chemical transformations where the tissues have specialized functions), and the genome, which is the basis of everything, via the molecules of life (DNA, RNAs, and proteins).

## Master Class Program About the Basis of Biochemistry and Molecular Biology and Rationale

Drawing up this program necessitates bearing in mind the students it is aimed at. In Europe in general, and in Spain in particular, access to the university degree course in medicine is extremely hard. The mark required in the university entrance exams is always very high, though it varies slightly from university to university. Given this, those students who follow the first-year course of BMB should, theoretically, have a high academic formation. All come from secondary education or high school, and their conceptual understanding of biology, general chemistry, and organic chemistry can vary, depending on the teaching received, the time dedicated to each subject, and the particular interest of the student.

The characteristics mentioned above warrant the inclusion of the basic concepts of BMB in the program to be taught to undergraduates in medicine. This program has evolved and developed over the years. The contents I propose do not really differ from those included in any current textbook, which are usually good. The conceptual content accompanying each section is greatly summarized for reasons of brevity and synthesis. The content has been designed considering the teaching and learning of the chemical molecular structure and, as the students are future doctors, including a few brief clinical reviews so that the students can appreciate how the content of each chapter affects a certain aspect of a disease or its treatment. By analyzing biochemical concepts in the context of a disease, the students learn why these concepts are relevant for human life and what happens when biochemistry becomes uncontrolled. I emphasize the term brief clinical reviews because the central purpose of our discipline is to teach the molecular structures and their transformations, always bearing in mind that their final aim is to maintain human life. Later on, in more advanced classes, professionals from national public hospitals entrusted with teaching medical and surgical subjects will explain these diseases in more detail from the clinical viewpoint. Table 1 shows the program, bearing in mind the creation of the European Higher Education Area that came into force for the academic year 2010–2011. BMB is a compulsory annual subject and forms a part of the basic education module. Given during the first course of the degree in medicine, it is divided into two semesters and at the Faculty of Medicine of Malaga University is assigned 15 credits in the European Credit Transfer and Accumulation System (ECTS) [6–8]. Six of these correspond to the first semester and nine to the second. Note that in this program I only include the number of hours of class attendance dedicated to the master lesson. The program consists of an introductory chapter, or prologue, and 24 other chapters, organized in three sections.

**Table 1:** Teaching program for biochemistry and molecular biology in accordance with the protocols of adaptation to the European Higher Education Area and European Credit Transfer and Accumulation System (ECTS)

Chapters and denomination		
	ECTS	Master class (time in hours per semester)
First semester	6	40 h
Section I. Molecular nutrients of life: structure and catalysis		
Preface: biochemistry and the unity of life		
1. Water and acid-base balance		
2. Amino acids peptides and proteins. Protein research: basic concepts		
3. The three-dimensional structure of proteins		
4. Protein function		
5. Enzymes		
6. Carbohydrates and glycoconjugates		
7. Lipids		
8. Biological membranes and transport		
9. Signal transduction		
10. Nucleotides and nucleic acids		
Second semester	9	60 h
Section II. Processing and storage of energy from molecules of life: bioenergetics and metabolism		35 h
11. Bioenergetics and basic concepts of metabolism		
12. Digestion and absorption of nutrients		
13. Glycolysis and gluconeogenesis		
14. The citric acid cycle		
15. Oxidative phosphorylation and photophosphorylation		
16. Metabolism of glycogen and pentose phosphate pathway		
17. Metabolism of fatty acids and lipids		
18. Metabolism of nitrogen compounds		
19. Hormone regulation and integration of metabolism		
Section III. Synthesis of the molecules of life: information pathways		25 h
20. DNA metabolism		
21. RNA metabolism		
22. Synthesis and protein turnover		
23. Regulation of gene expression: basic mechanisms		
24. Information technologies based on DNA. Genomic, proteomic, and metabolomics		

The first semester starts with an introductory class, mentioning that living systems need a very limited repertory of atoms and molecules plus four main types of biomolecules (proteins, carbohydrates, lipids, and nucleic acids). Mention is also made of how membranes delimit the cells, the biochemical functions confined within the cellular organelles that process and select proteins and exchange materials with the environment, and the central dogma of molecular biology. This chapter is then concluded highlighting that failures in the function of organelles can give rise to severe diseases, such as familial hypercholesterolemia and Tay-Sachs disease.

Section I of the program (chapters 1–10) introduces the student to structural biochemistry and enzymology. Explanations are also given about the cornerstones of nucleic acids, nucleotides, their structure, and physical and chemical properties, emphasizing their multifunctionality as mediators of energy exchange and chemical signals.

Looking at this section from the clinical viewpoint will enable the student to understand the basic principles of acidosis and alkalosis via pH buffering mechanisms. In relation to the structure and function of proteins, we can explain the disorders produced by the accumulation of incorrectly folded proteins (like the spongiform encephalopathies), the problems related with connective tissue and structural proteins, the hemoglobinopathies, the actions of drugs, the dosage of certain drugs based on enzyme inhibition, and the monitoring of tissue damage by measuring the blood enzyme levels. Carbohydrates, lipids, and proteins, the basic fuels of nutrition in man, are obtained from food and stored in specific depots. The study of these biomolecules will give the students’ knowledge about nutrition, enabling them to design and prescribe suitable diets. The membranes constituting the essential structural component of the cell facilitate the exchange of information via a process of biosignaling and transporting substances between the cells and the extracellular medium. These last chapters are very important to be able to understand how cells function and regulate their own proliferation, which forms the basis of understanding cancer and its treatment, as well as those disorders affecting specific cellular organelles.

The second semester encompasses sections II and III. About two thirds of these (chapters 11–19) are dedicated to the study of metabolism, a process undertaken by a very integrated set of reactions called intermediary metabolism. As mentioned earlier, what a graduate in medicine should know at the end of their studies is based on the suggestions proposed by Epp [5]. Although these suggestions may appear old-fashioned, I believe they are nevertheless very suitable to approach the study of the different metabolic pathways that comprise the intermediary metabolism. These proposals are:

1. Understanding the purpose of the pathway
2. Analysis of the major stages
3. Transformations undergone by the molecular skeleton component of the pathway under study
4. Study of the energy exchanges in the pathway
5. Study of the most important reactions
6. Study of the control mechanisms of the pathway
7. Short clinical concepts or applications relating to the diseases associated with each of the metabolic pathways

In stage 7, Epp suggests a very detailed thorough analysis of the various stages of the pathway. I have substituted this particular point with giving a series of brief clinical reviews in most of the chapters.

The students are taught that converting a meal into biochemical cellular compounds necessary for subsistence requires understanding the basic metabolism of fuels, critical for understanding the normal working of the human being, and that recognizing the anomalies of this metabolism will enable the diagnosis and treatment of a wide variety of disorders. The contents of this section will help the students understand the main principles of energy production, with special emphasis on the study of ATP, the universal free energy currency that transfers this energy from the processes that produce it to those that use it. We then study the process of food digestion that prepares the large biomolecules (carbohydrates, lipids, and proteins) in their basic forms for use by the metabolism. The main product of digestion of dietary carbohydrates is glucose, though some galactose and fructose are also produced. Study of the metabolism of glucose and other carbohydrates sets the scientific foundation for the treatment of diabetes and other carbohydrate disorders and shows the importance of the formation of glucose from other compounds apart from carbohydrates, mostly in the liver with the process of gluconeogenesis, and the formation of compounds that contain glucose in all the body tissues, like glucoconjugates.

Most of the carbons from the products of digestion (glucose, fatty acids, glycerol, and amino acids) are, in the end, converted into acetyl-CoA. These reactions produce ATP. Acetyl-CoA is oxidized in the citric acid cycle. CO_2_ is released and electrons are transferred to NAD^+^ and FAD^+^ to produce NADH and FADH_2_, vitamin cofactors that transfer the electrons to O_2_ via the electron transport chain. The energy from this transfer of electrons is used to produce ATP by the process of oxidative phosphorylation. The study of these chapters will be useful clinically to understand the function of the vitamins and the mechanisms of interruption of energy production by toxins or deficiencies. Consideration is also given to the toxicity of oxygen-derived radicals and their relation with disease, causing cell damage if the mechanisms of their synthesis and use are not carefully controlled.

Glycogen is the main form of storing glucose in humans. Mention is made that muscle glycogen is used to generate ATP for muscle contraction and that hepatic glycogen is used to maintain glycemia during fasting or exercise. Special mention is made about the maintenance of glycemia being an important function of the liver, as this organ produces glucose either by glycogenolysis or by gluconeogenesis. But not all glycogen metabolism is related with energy requirements. The final product of glycogen decomposition—glucose 6 phosphate—can also be metabolized via the phosphate pentose pathway, which produces NADPH (used in fatty acid synthesis, drug metabolism, and reduction of glutathione to diminish oxidative stress) and ribose-5-phosphate for the production of nucleotides. From the clinical viewpoint, this chapter describes the consequences and the origin of diseases related with glycogen storage in the liver and muscle tissue.

In the chapter devoted to lipid metabolism, we study how the triacylglycerols, which constitute the main source of energy for the organism, are obtained from food or synthesized, mainly in the liver. They are transported in the blood as lipoproteins and stored in the adipose tissue. During fasting, fatty acids from the triacylglycerols are oxidized by various tissues to produce energy. In the liver, the fatty acids are converted into ketone bodies that are oxidized in tissues like the brain, muscle, or kidneys. Mention is made of how the eicosanoids (prostaglandins, thromboxanes, and leukotrienes) are synthesized from polyunsaturated fatty acids. The main clinical aims of this topic are to understand the basic mechanisms of lipid disorders and their treatment, understand obesity and weight loss, and become familiar with the basics of the drugs that act on the eicosanoids.

Finally, the undergraduates study nitrogen metabolism with the degradation of the amino acids. The excess amino acids that are unnecessary for the biosynthesis of proteins cannot be stored or excreted and are therefore used as fuel. Firstly, the amine group is eliminated and converted into urea in the liver and finally excreted via the kidneys. Although urea is the main nitrogenized product of excretion, nitrogen is also excreted as NH4^+^, uric acid, and creatinine. In the postprandial state, the liver can convert the carbonated skeletons of amino acids into fatty acids and glycerol, which form the triacylglycerols of VLDL. In the fasting state, muscle protein is degraded, providing the blood with amino acids, and the liver converts their carbons to glucose or ketone bodies. Mention is made that the essential amino acids need to be obtained from food and that the nonessential amino acids can be synthesized by humans as the carbonated skeletons of amino acids can be obtained from glucose via the intermediaries of the citric acid cycle. Later, study is made of how the amino acids are used as building blocks to synthesize a large number of other important molecules containing nitrogen as the purine and pyrimidine bases, glutathione, phosphate creatine, biogenic amines, and porphyrins. The main clinical aim of this chapter is to understand protein metabolism, nitrogen bases, and amino acids. Products generated by protein metabolism include enzymes, DNA, RNA, and hormones. Their anomalies and deficiencies can cause a multitude of disease processes, such as anemia, liver or kidney disease, and congenital errors.

The study of the intermediary metabolism concludes with a topic devoted to metabolic integration. A summary is then made of the four main tissues that have a dominant role in energy metabolism: liver, adipose tissue, muscle, and brain, as well as the metabolic effects of insulin and glucagon, the main metabolic pathways, and the underlying regulatory mechanisms, the understanding of which has improved as a result of progress in identifying the signaling pathways. These pathways have often been used as pharmacological targets and highlight the impressive therapeutic progress in such fields as oncology. This last chapter enables the students to consolidate all they have studied about metabolism and its various pathways and provides an overall view that makes it easier to understand the idea of the intermediary metabolism.

Section III (chapters 20–24) deals with the structure of the macromolecules of life that contain information: DNA and RNA. Their study occupies one third of the program of master classes during the second semester. The order of explaining the topics basically follows the order in which they were discovered, which also coincides with the direction of flow of the information.

We start by studying the nucleic acids, concentrating on their structure. Drugs like cisplatin and others, used in chemotherapy against cancer, alter the structure of DNA, inhibiting replication and transcription of cancer cells. Hereditary information is coded in the DNA, which in eukaryotes is found in the nucleus and, to a lesser extent, in the mitochondria. Genetic information is inherited and expressed. Inheritance occurs via the process of replication. The DNA progenitor chains act as templates for the synthesis of copies that pass on to daughter chains. Mutations that damage DNA can cause genetic alterations, such as cell proliferation and cancer. The repair mechanisms are sometimes able to correct the damaged DNA. Gene recombination promotes genetic diversity.

Gene expression requires two steps, transcription and translation. DNA is transcribed to form messenger RNA (mRNA), which is translated to produce proteins. Ribosomal RNA (rRNA) and transfer RNA (tRNA) intervene in the process of translation.

Proteins participate in the cell structure and function as enzymes, which determine the reactions that occur in the cells. Thus, proteins, which are products of genes, determine the aspect and behavior of cells. Gene expression is regulated by a large variety of mechanisms. Only a small part of the genome is expressed in any cell. The purpose is to introduce the basic concepts involved in the regulation of genes that encode proteins and how they intervene in causing human disease.

Recombinant DNA technology has been developed to study and manipulate genes, as well as for more precise diagnostic purposes. Gene replacement therapy has only been successful in a few cases, and efforts are currently being made to reduce the side effects. We end our travel around the information pathways examining the relation between the omics technologies. These technologies have an extraordinary potential to assess risk and for the early detection, diagnosis, stratification, and individualized treatment of certain human diseases. An explanation is given about the benefits of these technologies for risk assessment and the design of personalized treatment. The very fast advances being made in medicine relating to diagnosis, prognosis, and treatment make it necessary to continually review and update this subject. The main clinical purpose of this section is to understand the molecular basis of genetic mutations, the importance of DNA repair, and certain viral and bacterial infections. It is vital to study and understand protein synthesis and antibiotics directed against this synthesis in the prokaryotes, as well as in toxins. Finally, the students are taught that understanding the future of diagnostic medicine requires use of recombinant DNA technology for the testing and diagnosis of specific diseases.

It is important to note here that the contents of this program are very much in line with the objectives and competencies described in the third International Conference of the Association of Biochemistry Educators (ABE) [9]. Likewise, my program is very similar to the BMB course given at the Department of Biochemistry and Molecular Biology, University College of Human Medicine, Michigan State University, a participating institution at the sixth International Conference of ABE (2017, Clearwater Beach, FL, USA) [10]. Additionally, both the prerequisites of Michigan State University to access the BMB course and the development of their program are all similar to the situation set out in this monograph for Malaga University.

A number of generally very good textbooks are available to study the topics mentioned above. Nevertheless, professors who have been teaching courses for several years are aware that the students, especially those studying for degrees in medicine or other health sciences, have limited time to study or revise any particular topic. Consequently, as the students do not have the time to consult all these texts, and remembering that our particular subject is not the only one (though each professor usually considers his or her subject as the most important in the year or even in the whole degree course), I consider the texts by Lehninger [11] and Stryer [12] always worth consulting and those of Baynes et al. [13], Feduchi et al. [14], Ferrier [15], and Lieberman [16], among others, slightly easier for the student.

## Limitations Inherent to the Teaching and Learning of This Subject

### General Scientific Terminology

Throughout my career as a professor of BMB at the Faculty of Medicine, I have noticed that one of the first mistakes made by students of medicine that I have at least tried to correct is the term or denomination of the subject “biochemistry and molecular biology.” In most medical faculties in Spain, this subject is taught during the first year. Almost every day, I have had to remind the students to use the correct term for this scientific subject. It is not called “chemistry” but rather “biochemistry.” What is the difference between one and the other? Biochemistry is the chemistry of life, and life itself is chemistry. Although it is obviously not as easy as this, the more we know about the human structure and functions, the more we understand that everything comes down to interactions between molecular structures. Really understanding the human body and maintaining its healthy status and quality of life necessitates understanding some of the basic facts about biochemistry, the “science of life.”

Once the students finally obtain their degree in medicine and start their clinical practice, one of the main objectives of the profession is very often the “maintenance of life.” Once this has been achieved, it is necessary to make sure the patient has the best possible chance of a good quality of life. It is here that medicine again relies on the pharmacopeia together with the other disciplines involved in the health sciences (nursing, physiotherapy, psychology, podiatry, odontology, etc.), where knowledge of BMB is also necessary, principally biochemistry.

### Understanding and Memorizing the Chemical Structures, Their Transformations, and Interactions

Another great problem when teaching this scientific subject concerns requiring the medical students to fully understand and memorize the molecular structures that form the basis of the material involved. This requires the cohesion and agreement of all the teaching staff in the department who give the theoretical classes to medical undergraduates, a criterion that is not easy to achieve given that the principle of freedom for each head of subject can affect this requirement. As a professor teaching BMB, I agree in general with Epp [5] that a certain degree of memorizing is needed to study and understand this subject. This, though, is not so difficult if the student has a good foundation in chemistry and biology from school. I understand that we cannot require the students to know all about certain very complex structures, such as vitamin B_12_, some coenzymes, and even the cytochromes that form part of the electron transport chain. Nevertheless, the student must be made to think about how the chemical structure of each of the cell components is intimately related with the cell function.

### Assessment of What Has Been Learned

In both the original degree course and in the current course for a degree in medicine, there are hardly any development-type exams in BMB. One reason is the large number of students (as occurs in our faculty). Accordingly, multiple choice exams are considered to be more objective to assess the students’ knowledge. Furthermore, the teaching process over the 6 years of the degree course is principally designed for the newly qualified doctors to obtain a post in a public hospital via the medical internship access system known as MIR (médico interno residente), the exams for which are multiple choice. Accordingly, the students do not have to discourse on any type of metabolic pathways or cycles and therefore do not study or attempt to retain a molecular structure. At the end of their university degree course, the students may have a fantastic mark in a subject but without knowing much about the molecules of glucose, a simple amino acid side chain or a fatty acid, and the compounds whose chemical molecules are not really very complicated. With this type of exam, it is, unfortunately, very difficult to include questions involving the molecular structures or development of a not too complicated metabolic pathway like glycolysis. Advances in new information and communication technologies now enable us to include in the exam at least a chemical structure to be recognized, a metabolic reaction for the students to indicate the enzyme responsible for the transformation of the substrate into the product, or the allosteric or hormone regulators that modulate a biochemical reaction.

## Conclusion

At the end of the university degree course in medicine, what a physician should know and understand about BMB is outlined in the program, divided into 3 main subject areas, with a total of 24 chapters. Learning BMB requires a sound basis in chemistry and biology, obtained during their school years, plus a certain degree of memorization. Memorizing the chemical structure of a biomolecule or a metabolic pathway involves understanding the strategy used by cells, as the basic component of an organ or tissue, to achieve a goal, which is none other than the maintenance of life and, in the last instance, of health, an inherent objective of any medical professional.
